# Can proactive support prevent unscheduled care? A controlled observational retrospective cohort study in cancer patients in Scotland

**DOI:** 10.1186/s12913-024-10923-2

**Published:** 2024-04-12

**Authors:** Austyn Snowden, Jenny Young, Jan Savinc

**Affiliations:** School of Health and Social Care, Edinburgh Napier University, Edinburgh, EH11 4BN Scotland

**Keywords:** Information management < BIOTECHNOLOGY & BIOINFORMATICS, Organisation of Health Services < HEALTH SERVICES ADMINISTRATION & MANAGEMENT ONCOLOGY, SOCIAL MEDICIN, Holistic Needs Assessment (HNA)

## Abstract

**Introduction:**

Preventative spend is a global health and social care strategy. Improving Cancer Journeys (ICJ) is a proactive, holistic, multidisciplinary project consistent with this agenda, currently being rolled out across Scotland and parts of UK. ICJ helps people with cancer access whatever support they need to mitigate their most pressing concerns. This study hypothesised that ICJ service users should subsequently use less unscheduled care than matched cohorts not using ICJ.

**Methods:**

Retrospective observational cohort study using linked national datasets. *N* = 1,214 ICJ users in Glasgow were matched for age, sex, deprivation, cancer type, stage, and diagnosis year to two control groups: 1. Cancer patients from Glasgow before ICJ (pre-2014), 2. Cancer patients from rest of Scotland during study period (2014–2018). Cancer registrations were linked for 12-month baseline and study periods to: NHS24 calls, A&E admissions, inpatient hospital admissions, unscheduled care, number & cost of psychotropic prescriptions. Per-person mean service uses were compared between groups.

**Results:**

There was a significant increase in NHS24 calls in the ICJ group (0.36 per person vs. -0.03 or 0.35), more and longer A&E attendances in ICJ (0.37 per person vs. 0.19 or 0.26; 2.19 h per person vs. 0.81–0.92 h), more and longer hospital admissions in ICJ (4.25 vs. 2.59 or 2.53; 12.05 days vs. 8.37 or 8.64), more care pathways involving more steps in ICJ (0.77 spells vs. 0.39 or 0.57; 1.88 steps vs. 1.56 or 1.21), more psychotropic drug prescriptions and higher costs in ICJ (1.88 prescription vs. 1.56 or 1.21; £9.51 vs. £9.57 or £6.95) in comparison to both control groups.

**Discussion:**

ICJ users sourced significantly more unscheduled care than matched cohorts. These findings were consistent with much of the comparable literature examining the impact of non-health interventions on subsequent health spend. They also add to the growing evidence showing that ICJ reached its intended target, those with the greatest need. Together these findings raise the possibility that those choosing to use ICJ may also be self-identifying as a cohort of people more likely to use unscheduled care in future. This needs to be tested prospectively, because this understanding would be very helpful for health and social care planners in all countries where proactive holistic services exist.

**Supplementary Information:**

The online version contains supplementary material available at 10.1186/s12913-024-10923-2.

## Introduction

Prevention has been a goal of health and social care policy for decades. Prevention policy refers broadly to government actions to intervene early in people’s lives, to reduce their need for acute and reactive services [1]. Earlier, targeted interventions should ideally mitigate more expensive interventions later. This was one of the hopes for ‘Improving Cancer Journeys’ [2].

It is widely acknowledged that any cancer diagnosis can have physical, psychological, social and practical impact beyond the individual, requiring a multidisciplinary response [3, 4]. In 2014, a multidisciplinary, multi-partner service called ‘Improving the Cancer Journey’ (ICJ) was created to support people in Glasgow, Scotland. The aim of ICJ was to meet the wider, holistic needs of people newly diagnosed with cancer in a proactive way [5]. The service invited all people diagnosed with cancer to have a holistic needs assessment (HNA), facilitated by specialist employees called ‘link officers’ [2]. The HNA process helped to identify the patient’s most pressing physical, social, emotional, or practical concerns so that tailored individualised interventions could then be agreed to help. Following agreed interventions, the HNA process was repeated until the patient’s concerns had been met and the link officer was no longer needed.

From 2015 to 2020, 6,130 people used ICJ. Of those, over 77% came from the most deprived areas, Scottish Index of Multiple Deprivation (SIMD) quintiles 1 & 2 [6]. On accessing the service, users baseline health related quality of life scores were among the lowest reported in the cancer literature [7], showing that the service was being used by those with considerable need. The most common concerns were housing, money, and transport. Following ICJ intervention these concerns were reduced to a level that was personally manageable [8]. Service user interviews showed that ICJ worked by helping people to self-manage problems before they instead became unmanageable, and having access to an ‘expert’ to guide them through an unfamiliar process was seen as invaluable [9].

Five interrelated elements explained why ICJ succeeded where it did:

1. Health and social care partners worked towards the same goals,
2. The HNA system facilitated joint working,
3. The link officers were valued by multidisciplinary colleagues,
4. Leadership at all levels was strong, and.
5. ICJ continually improved by embedding findings from its ongoing evaluation [5].

These elements were important to understand because in 2019 the Scottish Government match funded Macmillan Cancer Support to fund Transforming Cancer Care, a national roll out of ICJ to 36 new projects around Scotland [10].

Part of its appeal was ICJs apparent coherence with the principle of preventative spend. Service users had reported that ICJ had helped them manage problems ‘before they arose’ [11]. Users described feeling more in control, more organised [5]. It would follow that a reduction could be expected in use of subsequent unscheduled services such as A&E, because they are designed to manage *dis*organised, emergency care. Further, despite the mixed methods evidence cited above, none of it included causal evidence such as controlled studies. If a controlled study could demonstrate savings in unscheduled care, it would further validate the existing observational evidence for proactive holistic care.

The aim of this study was to establish whether ICJ service users used less unscheduled care in comparison to matched samples of controls.

### Ethics

Ethical permission to conduct the study was granted by the West of Scotland Research Ethics Committee (WoSREC: 15/WS/0199).

## Methods

Retrospective controlled cohort study using data linkage to connect routinely collected data held by ICJ service with hospitalisation, cancer registration prescription and unscheduled care service data held by Public Health Scotland.

### Cohorts & matching

The intervention group comprised a subset of users of the ICJ service during the period 2014–2018, aged 25 or above, residing in Glasgow at the time of their cancer diagnosis. In addition to the intervention group of ICJ users, two additional cohorts were defined for comparison, one group of cancer patients residing in Glasgow using services prior to the start of ICJ (2011–2013) and another contemporaneous group (using services during 2014–2018) residing in the rest of Scotland (ROS). The ICJ service was not available prior to 2014 in Glasgow, nor in the rest of Scotland during the evaluation phase, so the effect of the ICJ service could be estimated as the difference between the ICJ group and the other two cohorts.

Comparison cohort members were defined as exact matches to the intervention group on an individual level, on age group (25–34, 35–44, 45,54, 55,64, 65,74, 75+), sex assigned at birth, postcode-derived Scottish Index of Multiple Deprivation vigintile (SIMD; Scottish Government, 2016), year of diagnosis, and cancer type (lung, prostate, breast, bowel, or ‘other’). In addition, controls were excluded if they had a diagnosis of melanoma or other skin malignancy (C43-C44), if they had a second cancer diagnosis prior to the date of their matched intervention group members’ ICJ assessment, or if they were deceased prior to the 12-month service usage period, defined relative to their matched intervention group member (see Baseline and study periods section below). Of the approximately *N* = 4,200 users of ICJ Glasgow in the period 2014–2018, the final intervention group was composed of *N* = 1,214 individuals for whom matches were found in one or both comparison groups. *N* = 1,034 matching controls were found in Group 2 (pre ICJ Glasgow) and *N* = 1,108 matching controls were found in Group 3 (ROS), with 87% intervention group having a match in both.

### Baseline and study periods

To compare the effects of the availability of the ICJ service, a “baseline” period of service usage was defined, 12 months prior to every individual’s cancer registration date in SMR06. This was compared to the service usage during a period concurrent with ICJ usage, which was defined as the 12 months starting from the first ICJ assessment for ICJ users, or as the 12 months starting from the equivalent date relative to cancer registration for controls (for example, if an ICJ user had their first ICJ assessment 3 months after cancer registration, their matched control’s ICJ-concurrent period started 3 months after their cancer registration.

### Data linkage

The eDRIS team provided the data matching and linkage. In addition to the SMR06 (Cancer registrations) record, records from the following datasets were also linked: SMR01 (Scottish Morbidity Record; physical health admissions for in-patient and day cases); PIS (Prescribing Information System; Number & cost of community prescriptions); A&E admissions; NHS24 (24-hour health helpline) calls; CUPS (Unscheduled care pathways usage).

Linked data were available for the period 2011 to 2018. For the 7% of the pre-ICJ Glasgow control group who had cancer registrations in 2011, there was a risk of underestimating service usage during the baseline period as part of the baseline period may have started prior to 2011.

### Data analysis

We computed a proxy TNM-based stage using TNM staging, FIGO prior to and after surgery, and colorectal (Duke’s) staging variables from cancer registration data for lung, prostate, breast, and bowel cancers using algorithms published by the Detect Cancer Early programme [12]. Colorectal (Duke’s) stages A, B,C, D were considered equivalent to stages 1,2,3,4, respectively. FIGO stages were simplified to their numeric value (e.g. stage 2b was simplified to 2). Where an individual had multiple cancer registration entries for the same cancer type (lung, prostate, breast, bowel, or other), the highest stage was used. All other cancer types were grouped into ‘other’.

Service usage was defined as: number of NHS24 calls made; number of A&E admissions; time spent in A&E; inpatient hospital spells in hospital (SMR01 data); total days spent in hospital; number of unscheduled care (UC) pathway entries (Continuous Unscheduled care PathwayS—CUPS, refers to a series of UC contacts for a single person), number of steps in unscheduled care pathway entries; number of prescriptions for psychotropic drugs; cost of prescriptions for psychotropic drugs. Mean service uses per person were computed to compare the overall service usage of the groups. The 2016 version of the Scottish index of multiple deprivation (SIMD) was derived from postcodes of residence.

Descriptive statistics were computed for the sample characteristics and service usage measures. Differences between means (“change scores”) of service usage between baseline and ICJ-concurrent periods were computed for the groups and compared across groups using two-sided Welch’s t-tests.

Days in hospital were computed as the difference between discharge and admission dates plus one (so that discharges on the same day are treated as one day). The number of care pathways was the number of entries representing continuous spells in the unscheduled care pathway dataset; the number of steps was computed as the total of the individual services used as part of all care pathways for an individual (e.g. an NHS24 call followed by an ambulance use followed by an admission to A&E counted as 3 steps). Psychotropic drugs were defined as those belonging to Chaps. 4.1 Hypnotics and anxiolytics, 4.2 Drugs used in psychoses and related disorders, 4.3 Antidepressant drugs, 4.4 CNS stimulants and drugs used for attention deficient hyperactivity disorder, 4.11 Drugs for dementia, in the British National Formulary [13].

## Results

### Cancer stage

**Fig. 1:**
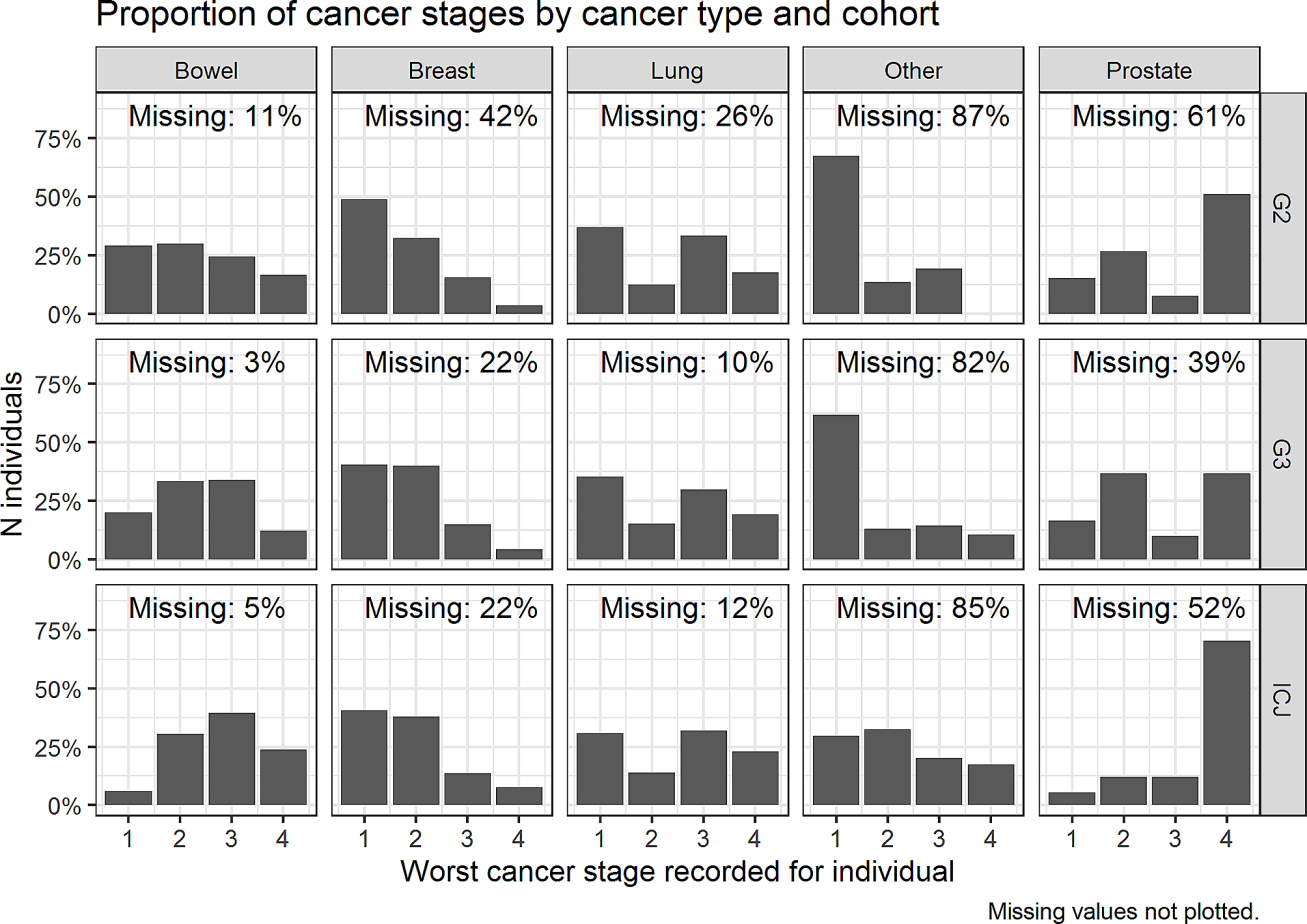
Distribution of cancer stages: frequency of stage by cancer & group. G2 = Pre-ICJ Glasgow group, G3 = ROS. Missing values not plotted. Note that some individuals had more than one cancer type recorded, and more than one record of the same type: the highest stage was used for each individual per type

Figure 1 shows the frequency of cancer stage according to type & study group, with Group 1 (G1) representing ICJ, Group 2 (G2) representing Pre-ICJ Glasgow group, Group 3 (G3) Rest of Scotland (ROS). Missing values were not plotted. Note that some individuals had more than one cancer type recorded, and more than one record of the same type, so the highest stage was used for each individual per type.

Table 1 shows the three groups matched by age, sex, and SIMD vigintile. It shows most cells to be within a percentage point of the original cohort. A descriptive summary of cancer stages by type can be found in Table 2 and collapsed by group in Table 3. There were high levels of missingness in cancer staging data, particularly for ‘other’ (82–87%) and prostate (39–61%) cancers. The levels of missingness were similar between the contemporaneous ICJ and ROS groups, and higher in the earlier pre-ICJ Glasgow group, probably reflecting differing availability of data over time. The particularly high level of missingness for the ‘other’ type probably reflects our use of staging variables for the major cancer types that did not capture staging data for other types of cancer.

**Table 1:** Demographics. Age, sex and SIMD quintile by group

	Group 1: ICJ	Group 2: Glasgow, pre-ICJ	Group 3: ROS
**N**	1214	1034	1108
**Sex**			
Female	693 (57.1%)	591 (57.2%)	640 (57.8%)
Male	521 (42.9%)	443 (42.8%)	468 (42.2%)
**Age**			
25–34	43 (3.5%)	32 (3.1%)	32 (2.9%)
35–44	90 (7.4%)	68 (6.6%)	69 (6.2%)
45–54	244 (20.1%)	204 (19.7%)	224 (20.2%)
55–64	381 (31.4%)	334 (32.3%)	360 (32.5%)
65–74	308 (25.4%)	262 (25.3%)	287 (25.9%)
75+	148 (12.2%)	134 (13.0%)	136 (12.3%)
**SIMD (1 = most deprived)**			
1	689 (66.6%)	755 (62.2%)	681 (61.5%)
2	156 (15.1%)	197 (16.2%)	180 (16.2%)
3	90 (8.7%)	121 (10.0%)	113 (10.2%)
4	71 (6.9%)	97 (8.0%)	90 (8.1%)
5	28 (2.7%)	44 (3.6%)	44 (4.0%)

**Table 2:** Descriptive summary of cancer stage by cancer type showing proportion of missing values. N refers to the number of non-missing values

	ICJ			Glasgow, pre-ICJ			ROS		
Cancer type	N	Missing	Mean [95% CI]	N	Missing	Mean [95% CI]	N	Missing	Mean [95% CI]
Bowel	180	5%	2.81 [2.68–2.94]	127	11%	2.28 [2.10–2.47]*	171	3%	2.39 [2.25–2.53]*
Breast	249	22%	1.88 [1.77–1.99]	171	42%	1.74 [1.61–1.86]	226	22%	1.83 [1.72–1.94]
Lung	178	12%	2.47 [2.30–2.64]	115	26%	2.32 [2.10–2.53]	149	10%	2.33 [2.15–2.52]
Other	76	85%	2.26 [2.01–2.50]	52	87%	1.52 [1.30–1.74]	78	82%	1.74 [1.50–1.98]
Prostate	74	52%	3.47 [3.26–3.68]	53	61%	2.94 [2.62–3.27]	79	39%	2.67 [2.42–2.93]*

* indicates that the mean was different from the ICJ group at *p* <.025

**Table 3:** Descriptive summary of cancer stage collapsed over groups

	N	Mean [95% CI]	SD
Group 1: ICJ	757	2.43 [2.28–2.59]	2.22
Group 2: Glasgow	518	2.10 [1.90–2.30]	2.28
Group 3: ROS	703	2.16 [1.99–2.33]	2.31

In the available data, cancer stage was higher in the ICJ group (M = 2.43, 95% CI 2.28–2.59) than either the pre-ICJ Glasgow (M = 2.10, 95% CI 1.90–2.30, *p* =.00959) or ROS groups (M = 2.16, 95% CI 1.99–2.33, *p* =.0199), suggesting that people in the ICJ group had more severe cancers. The difference was due to higher stage scores on Prostate, Bowel & Other cancers in the ICJ group, whereas Breast & Lung cancer stages weren’t significantly different.

### Analysis

**Fig. 2:**
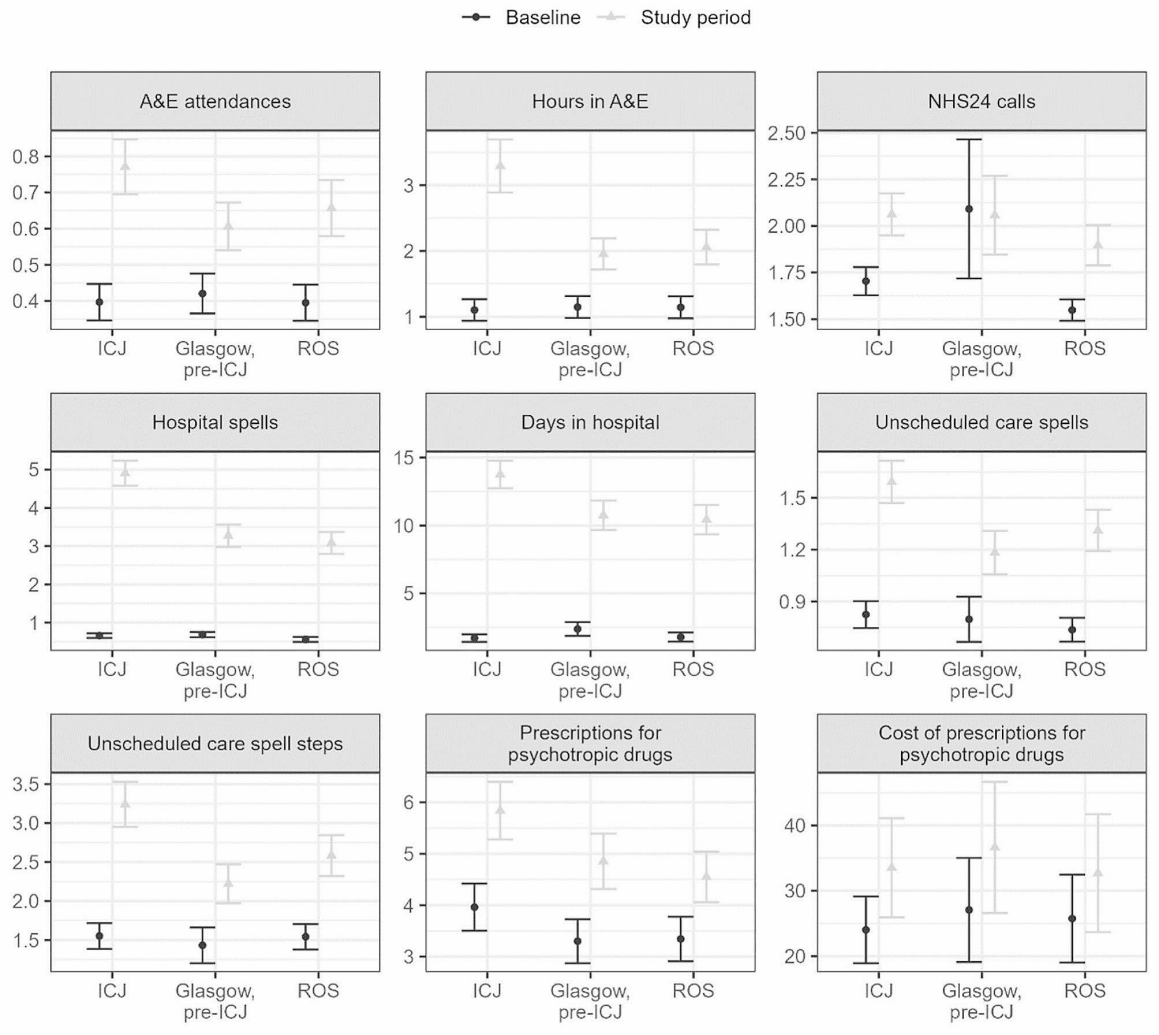
Service use means with 95% confidence intervals. Means were computed with entire group as denominator so non-users were included. Note that the scales are different between services. Baseline service use is shown in black and study period use is shown in grey

Table 4 contains descriptive summaries of service usage during the baseline and study periods. Table 5 shows the service usage change scores between baseline and study periods. Figure 2 illustrates mean service use measures during the baseline and study periods.

**Table 4:** Descriptive summary of service usage at baseline and study periods

	ICJ		Glasgow		ROS	
Mean [95% CI]	Baseline	Study period	Baseline	Study period	Baseline	Study period
A&E attendances	0.40 [0.35–0.45]	0.77 [0.70–0.85]	0.42 [0.37–0.48]	0.61 [0.54–0.67]	0.40 [0.35–0.45]	0.66 [0.58–0.73]
Hours in A&E	1.10 [0.94–1.27]	3.29 [2.89–3.70]	1.15 [0.98–1.31]	1.95 [1.72–2.19]	1.14 [0.97–1.31]	2.06 [1.79–2.32]
NHS24 calls	1.70 [1.63–1.78]	2.06 [1.95–2.17]	2.09 [1.72–2.46]	2.06 [1.85–2.27]	1.55 [1.49–1.61]	1.90 [1.79-2.00]
Hospital spells	0.66 [0.60–0.72]	4.91 [4.58–5.24]	0.69 [0.62–0.76]	3.27 [2.98–3.56]	0.56 [0.49–0.62]	3.08 [2.80–3.37]
Days in hospital	1.71 [1.43–1.99]	13.76 [12.74–14.78]	2.38 [1.87–2.89]	10.75 [9.67–11.83]	1.79 [1.45–2.12]	10.43 [9.35–11.51]
Clinical pathways	0.82 [0.75–0.90]	1.59 [1.47–1.72]	0.80 [0.67–0.93]	1.18 [1.06–1.31]	0.74 [0.67–0.81]	1.31 [1.19–1.43]
Clinical pathway steps	1.55 [1.39–1.72]	3.24 [2.95–3.53]	1.43 [1.20–1.66]	2.22 [1.97–2.47]	1.54 [1.38–1.71]	2.58 [2.32–2.84]
Prescriptions for psychotropic drugs	3.96 [3.50–4.42]	5.84 [5.28–6.40]	3.30 [2.87–3.73]	4.85 [4.32–5.39]	3.34 [2.91–3.78]	4.55 [4.06–5.04]
Cost of prescriptions for psychotropic drugs	24.02 [18.92–29.12]	33.53 [25.94–41.12]	27.07 [19.11–35.03]	36.63 [26.58–46.69]	25.75 [19.01–32.48]	32.70 [23.67–41.72]

**Table 5:** Descriptive summary of service usage change scores between baseline and study periods

Mean [95% CI]	ICJ	Glasgow	ROS
A&E attendances	0.37 [0.28–0.46]*	0.19 [0.10–0.27]*	0.26 [0.17–0.35]*
Hours in A&E	2.19 [1.76–2.63]*	0.81 [0.52–1.10]*	0.92 [0.60–1.23]*
NHS24 calls	0.36 [0.22–0.49]*	-0.03 [-0.46-0.40]	0.35 [0.23–0.47]*
Hospital spells	4.25 [3.91–4.58]*	2.59 [2.29–2.89]*	2.53 [2.23–2.82]*
Days in hospital	12.05 [10.99–13.11]*	8.37 [7.17–9.56]*	8.64 [7.51–9.77]*
Clinical pathways	0.77 [0.62–0.91]*	0.39 [0.20–0.57]*	0.57 [0.44–0.71]*
Clinical pathway steps	1.69 [1.35–2.02]*	0.79 [0.45–1.13]*	1.04 [0.73–1.35]*
Prescriptions for psychotropic drugs	1.88 [1.15–2.60]*	1.56 [0.87–2.24]*	1.21 [0.55–1.86]*
Cost of prescriptions for psychotropic drugs	9.51 [0.37–18.65]*	9.57 [-3.25-22.38]	6.95 [-4.31-18.21]

* indicates a significant difference between baseline and study period service usage at *p* <.05

### NHS24

In the baseline period, 19% of ICJ users made one or more NHS24 calls, compared to 18% in Glasgow, and 20% in ROS. The increase in users for the ICJ group was largest during the study period to 31%, followed by Glasgow at 27% and ROS at 26%.

Mean NHS24 calls per person increased from baseline to the study period more in the ICJ (0.36, 95% CI 0.22.-0.49) than in the Glasgow group (-0.03, 95% CI -0.46-0.40; d = 0.39, *p* =.0016). Note that there was a very small number of high frequency callers in the Glasgow group at baseline and we observed no change between baseline and the study period. The difference in increased calls between the ICJ and ROS groups was not significant (0.35, 95% CI 0.23–0.47; d = 0.01, *p* =.84).

### A & E

In the baseline period, 27% of ICJ users attended A&E one or more times, compared to 27% in Glasgow, and 26% in ROS. In the study period, the proportion of A&E attenders increased to 41% in the ICJ group, compared to 35% in Glasgow and 34% in ROS.

A&E attendances increased from baseline to the study period more in the ICJ (0.37, 95% CI 0.28–0.46) than both the Glasgow group (0.19, 95% CI 0.10–0.27; d = 0.19, *p* <.0001), and the ROS group (0.26, 95% CI 0.17–0.35; d = 0.11, *p* =.0013).

Hours spent in A&E also increased from baseline to the study period more in the ICJ group (2.19, 95% CI 1.76–2.63) than both Glasgow (0.81, 95% CI 0.52–1.10; d = 1.38, *p* <.0001), and ROS (0.92, 95% CI 0.60–1.23; d = 1.28, p = < 0.0001).

### Hospital attendance

In the baseline period, 43% of ICJ users had one or more hospital admissions, compared to 42% in Glasgow, and 33% in ROS. In the study period, 77% of ICJ users had hospital admissions, followed by Glasgow at 68% and ROS at 67%.

Hospital spells increased from baseline to the study period more in the ICJ (4.25, 95% CI 3.91–4.58) than both in the Glasgow group (2.59, 95% CI 2.29–2.89; d = 1.66, *p* <.0001), and in the ROS group (2.54, 95% CI 2.23–2.82; d = 1.7, *p* <.0001).

Days in hospital also increased from baseline to the study period more in the ICJ (12.05, 95% CI 10.99–13.11) than both in the Glasgow group (8.37, 95% CI 7.17–9.56; d = 3.68, *p* <.0001), and the ROS group (8.64, 95% CI 7.51–9.77; d = 3.41, *p* <.0001).

### Unscheduled care pathways

In the baseline period, 46% of ICJ users had one or more care pathways recorded, compared to 42% in Glasgow, and 42% in ROS. In the study period, 61% of ICJ users had one or more care pathways recorded, followed by Glasgow at 51% and ROS at 53%.

The number of care pathways increased from baseline to the study period more in the ICJ (0.77, 95% CI 0.62–0.91) than both in the Glasgow group (0.39, 95% CI 0.20–0.57; d = 0.38, *p* <.0001), and in the ROS group (0.57, 95% CI 0.44–0.71; d = 0.19, *p* =.041).

The complexity of care pathways (number of steps) increased from baseline to the study period more in the ICJ (1.69, 95% CI 1.35–2.02) than both in the Glasgow group (0.79, 95% CI 0.45–1.13; d = 0.9, *p* <.0001), and in the ROS group (1.04, 95% CI 0.73–1.35; d = 0.65, *p* <.0001).

### Prescriptions for psychotropic drugs

In the baseline period, 95% of ICJ users had one or more prescriptions for psychotropic drugs, compared to 94% in Glasgow, and 94% in ROS. In the study period, 99% of ICJ users had one or more prescriptions for psychotropic drugs, followed by Glasgow at 98% and ROS at 98%.

The number of psychotropic drug prescriptions increased from baseline to the study period in both ICJ (1.88, 95% CI 1.15–2.60) and in the Glasgow group (1.56, 95% CI 0.87–2.24), but the increase was not significantly different (d = 0.32, *p* =.21); there was a significant difference in the increase in prescriptions between the ICJ and ROS groups (1.21, 95% CI 0.55–1.86; d = 0.67, *p* =.0075).

The cost of psychotropic prescriptions only increased between baseline and the study period in the ICJ group (9.51, 95% CI 0.37–18.65) but not the Glasgow (9.57, 95% CI -3.25-22.38) or ROS (6.95, 95% CI -4.31-18.21) groups; the differences in increased cost weren’t significant between ICJ and Glasgow (d=-0.061, *p* =.99) nor ICJ and ROS (d=-0.061, *p* =.99). The cost of psychotropic drug prescriptions was somewhat lower and less variable in the ICJ group at baseline.

## Discussion

It was hypothesised that ICJ users would subsequently require fewer unscheduled health services, having had more of their needs met by ICJ and the resulting referrals. Instead, the study revealed the opposite: a larger increase in NHS24 calls in the ICJ group than in ROS, more and longer A&E attendances in ICJ than in Pre-2014 and ROS, more and longer hospital admissions in ICJ than Pre-2014 and ROS, more care pathways involving more steps in ICJ than Pre-2014 or ROS, more psychotropic drug prescriptions in ICJ than in ROS, and a significant increase in psychotropic prescription costs in ICJ, which was not observed in Pre-2014 or ROS. This study showed that those provided with proactive and holistic support used more services, including unscheduled care, than those not.

Similar findings have been explained as a function of empowerment [14]. Empowered people might be expected to use more scheduled or routine services. Early feedback from ICJ users showed increased confidence as a function of the support received [15]. One of the consequences of increased confidence is confidence to access resources previously considered inaccessible [16]. However, higher levels of empowerment wouldn’t explain the higher levels of unscheduled care pathways, hospital admissions or A&E attendance. They are better explained as a function of illness complexity, severity, and unmet need.

The results of this study appear to run contrary to the almost universally positive observational findings about the project [5, 17]. They are however consistent with related attempts to quantify the impact of complex holistic, person-centred interventions on health spend. For example, a recent systematic review found no evidence to support social prescribing interventions [18]. The Evercare study [19] found personalised interventions to be very popular with patients, but they did not reduce hospital admissions. An evaluation of personal health budgets [20] found some improvement in quality of life, but no change in service use. Our study goes further to conclude that ICJ was associated with greater service use, not less. The idea that non-health interventions can reduce subsequent, more expensive health service use persists [21], but these claims often overreach the evidence [22].

It will be important to replicate this study prospectively as the ICJ programme is rolled out across Scotland [10], but these findings clearly show that health and social care planners should be wary of assuming early interventions in complex care will generate identifiable return on investment. Holistic care is complex and multifaceted, and unscheduled care is an essential element of that complexity. These services are funded to meet unplanned but *expected* need, the need for unscheduled and emergency care. People with advanced cancer from the most deprived areas should be expected to need these services [23].

Whilst minimising the use of expensive, unscheduled care is essential, understanding when it is likely to be necessary is also very helpful. ICJ was associated with people accessing more unscheduled services, not less, so *expecting* that should be the new norm as the programme goes nationwide. This is how policy and evidence should evolve, in tandem [24], and if replicable these findings should help future service planners allocate resources more strategically, turning unexpected care into expected care. It may be that the national rollout of ICJ is inadvertently identifying a cohort of people at higher risk of needing unscheduled care. This can be evaluated prospectively.

### Weaknesses and limitations

It is difficult to translate ‘statistical significance’ into ‘real world difference’. Clearly some service use increases (e.g. longer hospital stays) cost more than others (e.g. small increase in prescribing costs), so it could be argued that those with the largest cost implication are the most important. The key psychometric concept is the ‘minimally important clinical difference’ (MICD) [25], which refers to the smallest relative change in a measure that translates into a real world difference. There is no consensus on the best way of calculating MICD (please see Cook [26] for a critique), but there are conventions. For example, half a standard deviation is suggested as a ‘rule of thumb’ by some [27, 28], whereas others use the standard error [29]. All measures increased by more than one standard error (aside from the small decrease in NHS24 calls in G2). Not all measures met the SD criteria, but the more costly measures did. The number of hospitalisations (spells) were 1.23 times the standard deviation in ICJ (0.87 in G2, 0.85 in G3), and number of days in hospital were 1.04 times the SD in the ICJ group vs 0.64 in G2 and 0.72 in G3. These are real world differences.

It is possible that a subset of ICJ service users drove the increase in service usage. For example, despite the matching process, the distribution of staging scores in the ICJ group was higher for every cancer type (Table 2; Fig. 1), especially for prostate and ‘other’ types of cancer. For example, the mean staging scores for ‘other’ cancers were ICJ: 2.26, Group 2: 1.52, Group 3: 1.74, with over 60% of patients in Groups 2 & 3 having stage 1 recorded, as opposed to just over 25% in the ICJ group. Some cancers have been shown to be more distressing than others, and the way the groups were matched on broad cancer types, especially ‘other’ cancers, could have obscured such differences between specific cancer types. ‘Other’ cancers included some particularly distressing cancers, such as gastric [30] and cervical [31], which may not have matched against equivalently distressing diagnoses between groups. Further, the mean cancer stage was higher in the ICJ cohort for every cancer type (Table 2). In other words, the severity of disease was higher in the ICJ group, inferring that the group was more severely ill than the matched cohorts. If the ICJ cohort had more severe disease it follows that some may have been closer to death, and it is well established that cancer patients close to death use more unscheduled services [32].

The explanation that ICJ users had more severe levels of disease rests in part on differences in cancer staging data with high levels of missingness. As discussed, previous work evaluating ICJ showed high levels of need and low levels of self-reported quality of life [8], which supported our interpretation of the missing data. However, we did not control for comorbidities, which may have affected service usage in addition to the type & severity of cancer. Self-reported comorbidity data were collected by the ICJ service, but the equivalent could not be obtained for the comparison cohorts. Future studies could use linked historical hospitalisations or primary care data to estimate comorbidities.

Further, the matching procedure on age, sex, cancer type, diagnosis year, and a very granular deprivation measure (SIMD vigintile), resulted in approximately 29% of the ICJ service users in the period 2014–2018 being included in the analysis, which may have resulted in selection bias towards heavy service users. Additionally, individuals from the comparison group were excluded if they had a second cancer diagnosis at the time of their service use, which may have selected for lower service users.

The 12-month periods were chosen arbitrarily to define baseline and ICJ service-concurrent service usage periods. These were defined on an individual level, which means the results may have been confounded by seasonal and temporal effects, though we expected that these effects were small in the relatively short period 2011–2018. Although the ICJ service endeavours to assess users as soon as possible after their cancer diagnosis, the actual timing varies, which may have also confounded the results by capturing a period of heavy service use for the ICJ cohort but not for the comparison groups. A similar pattern would also be observed if waiting times for treatment were different between the cohorts, meaning that the same period might capture high service use for one person and not for another.

Finally, the intervention involved specialist link officers helping cancer patients who self-identified as needing support, and then receiving support with their most pressing physical, social, emotional, or practical concerns. Whilst clearly popular and beneficial to those who needed it, it is perhaps unrealistic to expect that this intervention would go on to mitigate the *need* for unscheduled care in future. In retrospect, the opposite makes more sense. Those people identifying themselves as needing help from ICJ are most likely going to be the same people for whom greater levels of unscheduled care should be *anticipated*. This finding needs to be replicated with a prospective study, because if true this information will be invaluable to service planners.

## Conclusion/implications for practice

ICJ was associated with significantly greater use of unscheduled care in the year following intervention, in comparison to two control cohorts matched for age, sex, deprivation, cancer type, stage, and diagnosis year. This study did not therefore find an easily identifiable return on investment from a health perspective. This can be explained as a combination of the unique nature of the ICJ cohort and the inherent limitations of matching. ICJ users previously recorded some of the lowest quality of life scores in the cancer literature. It follows that they would have a greater need for emergency and unscheduled care. Reflection on the matching process supported this view. There was higher average staging in the ICJ cohort, meaning cancer was more severe than in the comparator groups.

This study should therefore be replicated prospectively for three key reasons. First, it provided further evidence that ICJ was reaching its intended target, those with the greatest need. Given that targeting resources on those needing them most is the goal of health and social care spend, this is important to know, especially as ICJ is rolled out across Scotland and beyond. Second, this study should also help future planners better understand the limits to claims for preventative spend. Third and finally, those self-identifying as needing support from ICJ may also be identifying themselves as a cohort of people likely to use more unscheduled care in future. Understanding this should be helpful for health and social care planners across the world where similar proactive services exist.

### Electronic supplementary material

Below is the link to the electronic supplementary material.

Supplementary Material 1

Supplementary Material 2

## Data Availability

Data are not publicly available. The researchers only had access to pre specified anonymised datasets within a dedicated secure environment at NHS Scotland’s National Services ‘safe haven’. These anonymised datasets were not permitted to be exported. Interested researchers would need to apply to eDRIS for relevant access. Please see permissions algorithm in supplementary data, and contact authors a.snowden@napier.ac.uk or J.savinc@napier.ac.uk for further information.
