# Supplementary material for: Can proactive support prevent unscheduled care? A controlled observational retrospective cohort study in cancer patients in Scotland
Source: BMC Health Serv Res. 2024 Apr 12;24:457. doi: 10.1186/s12913-024-10923-2 (PMC11010331; doi:10.1186/s12913-024-10923-2)
Supplement: Supplementary file 1 — Supplementary Material 1 [file 12913_2024_10923_MOESM1_ESM.pdf]

How do we link the data?

NHS National Services Scotland

Data Provider A supplies personal identifiers to Indexing Team

→

Indexing team probability matches individuals to a population spine and creates anonymised ID

→

Indexing team returns anonymised ID to Data Provider A

→

Data Provider A matches anonymised ID on to payload data and removes personal identifiers

→

Data Provider A provides payload data to eDRIS team

→

eDRIS team links the data and

make anonymised data available to the researcher(s) in a secure environment

REPEAT WITH ALL DATA PROVIDERS INVOLVED IN STUDY

Indexing team send the Master Index File containing a Master Anonymised ID number + Data Provider A ID number + Data Provider B ID number + Data Provider C ID Number (+ …) to the eDRIS team

eDRIS team run the datasets sent by all data providers and Master Index file through a "linkage agent"

Linkage agent replaces Data Provider ID numbers in each dataset with the Master Anonymised ID number

Researcher receives data files with Master ID plus requested content data in safe haven environment

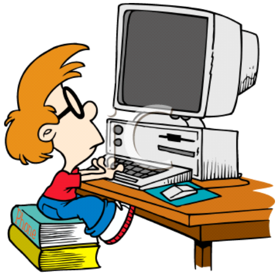

NHS National Services Scotland
